# On zombie papers

**DOI:** 10.1038/s41419-019-1450-3

**Published:** 2019-02-25

**Authors:** Enrico M. Bucci

**Affiliations:** 1 Sbarro Health Research Organization, c/o Temple University, 1900 N 12th St, Philadelphia, PA 19122 USA; 2 Resis Srl, Via Ivrea, 8/1, 10010 Samone, TO Italy

Retractions of scientific papers are valuable because they alert the scientific community that some specific scientific findings should no longer be considered valid, either because the underlying data are not solid or because the conclusions were unsupported by the data. In principle, once it is withdrawn, a paper is expected to be cited only with reference to its retraction, not in support of some research published later. However, at least in the last 30 years, this does not seem to be the case. We have witnessed the development of the so-called “zombie literature”: more often than expected, retracted papers continue to be cited in a positive way, as if they were still a valid science^1^. This is due to a variety of reasons, but chiefly has been attributed to the lack of clear and consistent flagging of retracted papers (often only the retracting note is available) and to the lack of a central repository, for all retracted papers, which could be used for checking the status of the literature cited by a given manuscript in preparation. While manuscript flagging has improved in recent years, it is only last year that the group led by Ivan Oransky released a massive retraction database^2^, which has been proposed to cross-check for retractions of the literature cited by each new manuscript^3^. This database seems to be still far from complete and it is dependent on the efforts of a private foundation, which relies on donations for its operations: an intolerable risk for such an important public resource. However, referees and editorial boards are strongly advised to make use of it for checking manuscripts’ references, and publishers are urged to develop automatic plugins or tools, which can facilitate this editorial task.

To clarify the risks of not doing so, here, I will highlight two cases of retracted papers where I was personally involved in uncovering the data manipulation.

In 2016, two papers^4,5^ authored by the group led by professor Federico Infascelli, purportedly “proving” the health risks of GMO food consumption, were retracted for containing manipulated images. The two papers had been used to campaign against GMO food; thus, the two retractions went viral in the scientific community, so that one of them was listed among the top 10 retractions occurring in 2016^6^. Despite the wide resonance of those retractions, we can read in a late 2018 paper^7^ discussing the risks for health of consuming GMO food the following words: “*A number of studies concerning this issue have previously been published concluding absence of any detrimental effects on human and animal health […]. Opposite opinions, however, do exist and are experimentally documented ([…], Tudisco et al., 2010)*”, where “Tudisco et al., 2010”^4^ refers to one of the two retracted papers.

The damages of reporting as valid data retracted for being manipulated, completely ignoring the retraction of that paper, are immediately apparent: a socially important issue, which has been already settled by science, is reported as still open to debate, in a journal published by a respected publisher, such as Wiley.

A more subtle effect is evident when considering negative citations of the two retracted papers, like, for example, in the case of a 2017 paper^8^ citing the original papers as examples of bad science. Despite the intention of the authors of the 2017 paper, simply discussing bad science and citing some examples will increase the citational impact of the authors found guilty of misconduct, i.e., retracted papers will continue to boost the bibliometric indexes of their authors even when they are simply pointed at for being fraudulent.

The second example I will refer to includes a larger sample of retracted papers to gain further insight on their citational impact. In 2011, I started investigating the scientific production of Alfredo Fusco, an Italian researcher in the field of preclinical oncology, after identifying seven papers containing manipulated images, which were flagged during an automated image screening^9^. The number of problematic papers identified during the investigation steadily grew up, before the news spread to the public in 2013;^10^ by the end of 2018, Fusco and his co-authors had retracted over 22 papers and published no fewer than 10 errata/corrigenda, which can be still considered a provisional figure for the overall impact expected as a consequence of the original investigation. Seven years after the appearance of the first retraction for image manipulations in 2013, there are some lessons to be learnt by the extensive literature correction caused by this case of misconduct: to what extent was the original damage truly accepted by the scientific community? How aware is this community of the retracted papers? A possible way to investigate this is to analyse how, and if, retracted papers are still cited by the scientific community, after the retractions occurred. In this case, one can refer to the data provided by Google Scholar: although the citations counted by this service are known to exceed those recorded by Scopus or Clarivate, they have the advantage of including also citations in a highly diverse range of scientific production, including, for example, PhD theses. For this reason, Google Scholar citations may best serve our aim, which is to measure the overall citational impact of the papers cited after their retraction. Ten publications were retracted in the period 2013–2017 (2018 retractions are too recent to count the citation); for eight of them, citational data are available at the time of preparation of this paper. Overall, those eight papers received 638 citations; on average, 18.6 ± 8% of the citations received by a retracted paper occurred at least 1 year after its retraction, adding up to 71 citations. The vast majority of these 71 citations are non-negative or positive (they are not criticising the original paper, without reporting its retraction), possibly in connection with the referring authors being not aware of the retractions.

Moreover, there is a further sinister phenomenon: authors of the retracted papers, which (by definition) are aware of the retraction, continue to cite those papers. Consider for example one of the notes of retraction by Fusco et al., appearing on May 12, 2016^11^. The retracted paper^12^ appears to be cited as reference n.9 by another paper published in 2018^13^. The retracted paper and the 2018 paper have one author in common: professor Alfredo Fusco, who of course cannot be unaware of his previous retraction. Thus, we have not only to fear “zombie papers”, but also Frankenstein doctors, who are resurrecting their withdrawn literature, pretending that their science was genuine and appropriating some more self-citations.

I hope at this point to have made some points clear:

1. There is an urgent need for reviewers and editorial boards to check the reference list of submitted manuscripts, to prevent the spreading of zombie literature.
2. We need central repositories of retracted papers, in order to facilitate the work of editorial boards and reviewers.
3. Ideally, this database should be under public control; anyway, it must be consistently funded (an interesting possibility would be to have it funded by the Committee for Publication Ethics—COPE).
4. We need to ensure that citations, referring to retracted papers when discussing their retraction, do not boost the bibliometric indexes of the retracting authors.
